# Medication adherence in Fabry patients treated with migalastat: Real world experience

**DOI:** 10.1016/j.ymgmr.2023.100976

**Published:** 2023-04-29

**Authors:** Eleonora Riccio, Oriana de Marco, Antonio Pisani

**Affiliations:** a Institute for Biomedical Research and Innovation, National Research Council of Italy (IRIB CNR), Palermo, Italy; b Chair of Nephrology, Department of Public Health, University Federico II of Naples, Italy

Dear Editors,

We read with great interest the paper by Müntze and colleagues [1], reporting an exceptionally high therapy persistence and adherence in 40 Fabry disease patients treated with the oral chaperone migalastat for 24 months. Authors reported ‘forgetfulness’ and ‘carelessness’ as main barriers to adherence, and supposed a possible selection bias in favour to particularly compliant patients agreeing in study population.

In this respect, here we report our ‘real-world’ single-center experience with therapy persistence/adherence in our population of 35 Fabry patients (15 males) treated with migalastat between 2017 and 2023. Out of these 35 patients, 10 (29%) discontinued therapy after a mean period of 20.4 ± 20.2 months. Main reasons for drug discontinuation were: estimated glomerular filtration rate < 30 mL/min/1.73 m^2^ (2 patients), pregnancy (1 patient), and self-chosen termination for the other 7 patients, with 2 patients reporting exacerbation of their depression, one reporting to feel better with therapy and believing it was no longer needed, 3 for carelessness for treatment, and one reporting to feel worse with the drug.

Moreover, therapy adherence evaluation of the 25 patients continuing therapy with migalastat (mean follow-up period of 37.4 ± 17.9 months), performed by MAQ [2], revealed that adherence was high in 21 (84%), and low in 4 patients (16%), mainly for ‘carelessness in medication intake’ (2 patients) and ‘stopping medication intake for feeling better’ (2 patients).

In conclusion, we observed that therapy persistence and adherence in our population of patients receiving migalastat were lower compared to those reported by Münze (29% vs 7.5%, and 84% vs 92.5%, respectively).

Main possible factors that might impact on therapy adherence are that some patients did not report beneficial effects with therapy for its long-term effect; on the contrary, other patients reported to feel good with the therapy, and stopped the drug believing it was no longer needed.

## Data Availability

No data was used for the research described in the article.
